# Fluid shear stress activates c-Src and promotes RANKL localization in the plasma membrane in osteoblast-like MC3T3-E1 cells

**DOI:** 10.1016/j.bbadva.2026.100185

**Published:** 2026-03-20

**Authors:** Takuma Matsubara, Anna Yoshimura, Shoichiro Kokabu

**Affiliations:** a Division of Biochemistry, Kyushu Dental University, Kokurakita-ku, Kitakyushu, Fukuoka 803-8580, Japan; b Kyushu Dental University Oral Medicine Innovation Center, Kokurakita-ku, Kitakyushu, Fukuoka 803-8580, Japan; c Division of Applied Pharmacology, Kyushu Dental University, Kokurakita-ku, Kitakyushu, Fukuoka 803-8580, Japan

**Keywords:** Mechanotransduction, c-Src, RANKL, Plasma membrane localization, Osteoclastogenesis

## Abstract

Receptor activator of NF-κB ligand (RANKL) produced by osteoblastic lineage cells is essential for osteoclastogenesis, yet RANKL can be sequestered in intracellular, lysosome-like compartments under basal conditions. How mechanical cues mobilize RANKL toward the cell surface remains poorly defined. Here, we tested whether fluid shear stress alters RANKL subcellular distribution in osteoblast-like MC3T3-E1 cells and examined the involvement of the non-receptor tyrosine kinase c-Src. MC3T3-E1 cells expressing fluorescently tagged RANKL were subjected to fluid shear stress, and RANKL localization was analyzed by microscopy and subcellular fractionation. Fluid shear stress increased c-Src activation (Tyr416 phosphorylation) and promoted redistribution of RANKL toward the cell periphery, accompanied by an increase of RANKL in the membrane fraction. Co-expression experiments showed spatial association of RANKL with c-Src at the cell periphery after shear stimulation. Moreover, constitutively active c-Src (Y527F) enhanced peripheral localization of RANKL even in the absence of shear stress. Together, these data support a model in which shear stress activates c-Src to facilitate RANKL localization from intracellular stores toward membrane-proximal regions, thereby providing a mechanistic link between mechanical cues and osteoblast-derived osteoclastogenic signaling.

## Highlights

● Fluid shear stress activates c-Src in osteoblast-like MC3T3-E1 cells.
● Shear stress redistributes RANKL toward the cell membrane fraction.
● l Constitutively active c-Src promotes RANKL redistribution without shear stress.
● Measured the events in Figure 3A and plotted them in a graph (Fig. 3B).

## Introduction

Bone undergoes continuous remodeling through bone resorption by osteoclasts and bone formation by osteoblasts [1]. This bone remodeling process is known to be regulated by mechanical stress. Osteoblastic lineage cells, such as osteoblasts and osteocytes, function as the primary mechanosensors. When osteoblastic lineage cells sense mechanical stress, bone formation signals are activated, promoting bone formation [2,3]. Simultaneously, the expression of RANKL, a cytokine essential for osteoclast differentiation and bone resorption [[4], [5], [6]], is induced in osteoblastic lineage cells [3]. However, for osteoblastic lineage cells to induce osteoclast differentiation, mere gene expression and protein synthesis of RANKL are insufficient; the synthesized RANKL must translocate to the plasma membrane and be presented on the cell surface as membrane-bound RANKL [7].

Under normal conditions, however, RANKL in osteoblastic lineage cells is localized mainly in intracellular lysosomal organelles (secretory lysosomes) and is barely detectable on the cell surface [8,9]. Therefore, to support osteoclast differentiation, RANKL stored in lysosome-like compartments within osteoblastic lineage cells must be intracellularly transported and presented on the surface via membrane fusion. Yet, how mechanical stimulation regulates this process has not been fully elucidated.

The non-receptor tyrosine kinase c-Src organizes the cytoskeleton, including actin filaments, to regulate cell migration and adhesion [10,11]. Additionally, c-Src is involved in the trafficking and secretion of intracellular vesicles such as lysosomes [12,13]. Furthermore, in various cells including osteoblasts, c-Src cooperates with cell adhesion molecules, such as integrins, to sense mechanical stress, whereby it becomes activated and reorganizes the cytoskeleton, including actin [14]. However, there have been no reports indicating that c-Src, upon sensing mechanical stress, is involved in the transport of RANKL stored in lysosome-like compartments.

In this study, we demonstrate that c-Src is activated by mechanical stress loading and promotes the translocation of RANKL stored in lysosome-like compartments to the plasma membrane.

## Materials and methods

### Cells

MC3T3-E1 cells (E1 cells) were purchased from American Type Culture Collection (ATCC, Manassas, VA) and cultured with alpha Modified Eagle's Medium (MEMα) from FUJIFILM Wako Pure Chemical Corporation (Tokyo, Japan) containing 10 % fetal bovine serum (FBS) from Sigma-Aldrich Co. LLC (St. Louis, MI).

### Plasmids

Src wildtype (WT) gene was purchased from Addgene (Cambridge, MA) and was mutated tyrosine 527 to Phenylalanine (Y527F) as previously described [10]. These cDNAs have undergone complete codon deletion followed by the addition of a Flag-tag (DNA sequence: 5′-GATTACAAGGACGACGATGACAAGTAG-3′) to the 3′ end (C-terminus) of the protein. Src WT was subcloned to pTagGFP2-N vector purchased from Evrogen (Moscow, Russia). RANKL gene was purchased from Dharmacon (Lafayette, CO) and subcloned to ptdTomato-N1 vector purchased from Takara Bio Inc. (Shiga, Japan).

### Transfection and observation

E1 cells were plated to 10,000 cells/cm^2^ in μ-slide 8well purchased from ibidi (Martinsried, Germany) or 35 mm φ glass bottom dish purchased from Matsunami glass Industry (Osaka, Japan). GFP, Src WT-GFP, Src Y527F-flag and RANKL-tdTomato plasmids were transfected with Screenfect™A purchased from FUJIFILM Wako Pure Chemical Corporation as the standard protocol. Cells were observed by fluorescence microscopy DeltaVision Elite purchased from GE healthcare Japan (Tokyo, Japan) as previously described [15].

### Immunofluorescence

E1 cells were fixed with 3.4 % formaldehyde and permeabilized with 0.2 % Triton X as previously described [15]. Subsequently, the cells were incubated with anti-flag anti body purchased from FUJIFILM Wako Pure Chemical Corporation / the Alexa Fluor 565-conjugated anti-mouse IgG and the Alexa Fluor 366-conjugated phalloidin purchased from Thermo Fisher Scientific, Inc. The subcellular localization of the indicated proteins was determined by DeltaVision Elite or BZ-X810 microscope.

### Fluid shear stress

E1 cells were 35 mm φ glass bottom dish or 35 mm culture dish purchased from Corning (Corning, NY). Dishes were put into Catch Burger purchased from Nepa Gene Co., Ltd (Chiba, Japan) and got a load on 12 dyn/cm^2^ shear stress with microtube pomp purchased from Icomes Lab Co., Ltd. (Iwate, Japan). The subcellular localization of the indicated proteins was determined by DeltaVision Elite. For measurements, RANKL located beyond half of the nuclear circumference in terms of cell diameter was considered as being localized in the periphery.

### Western blotting analysis

E1 cells were plated on 35 mm culture dish and got shear stress. To separate the cytoplasmic and membrane fractions, cells were first lysed with a lysis buffer (20 mM HEPES (pH 7.4), 150 mM NaCl, 1 mM EGTA, 1.5 mM MgCl_2_, 10 % glycerol, 0.05 % Triton X-100, 10 µg/mL aprotinin, 10 µg/mL leupeptin, 1 mM PMSF, and 0.2 mM sodium orthovanadate) to extract the cytoplasmic fraction [16]. The resulting pellets were then resuspended in RIPA buffer [0.1 % SDS, 1 % Triton X-100, 150 mM NaCl, 1 % sodium deoxycholate, 10 mM Tris, 5 mM EDTA, 10 µg/mL aprotinin, 10 µg/mL leupeptin, 1 mM PMSF, and 0.2 mM sodium orthovanadate] to extract the membrane fraction. For total protein extraction, cells were lysed directly in RIPA buffer. The supernatants were boiled in sample buffer, subjected to SDS polyacrylamide gel electrophoresis (SDS-PAGE), and transferred to a nitrocellulose membrane. The membrane was immunoblotted with the corresponding primary antibodies that are anti-Src antibody purchased from Merck Millipore (Darmstadt, Germany) and anti- phospho-Src (Tyr416) antibody purchased from Cell Signaling technology (Danvers, MA) and HRP-conjugated anti-mouse or anti-rabbit secondary antibodies purchased from Jackson immunoresearch laboratories, Inc. (West Grove, PA) and developed using Immobilon Western Chemiluminescent HRP Substrate purchased from Merck Millipore.

### Data analysis and statistics

Each data was checked for normality by the Shapiro-Wilk test. Differences between two groups were analyzed by the Student’s *t*-test to determine statistical significance. A value of *P* < 0.05 was considered statistically significant. Each sample data is represented by a dot and overlaid with a bar graph. Error bars in the bar graphs indicate standard error of mean (SEM).

## Results

### Alteration of RANKL localization by fluid shear stress

**Fig. 1:**
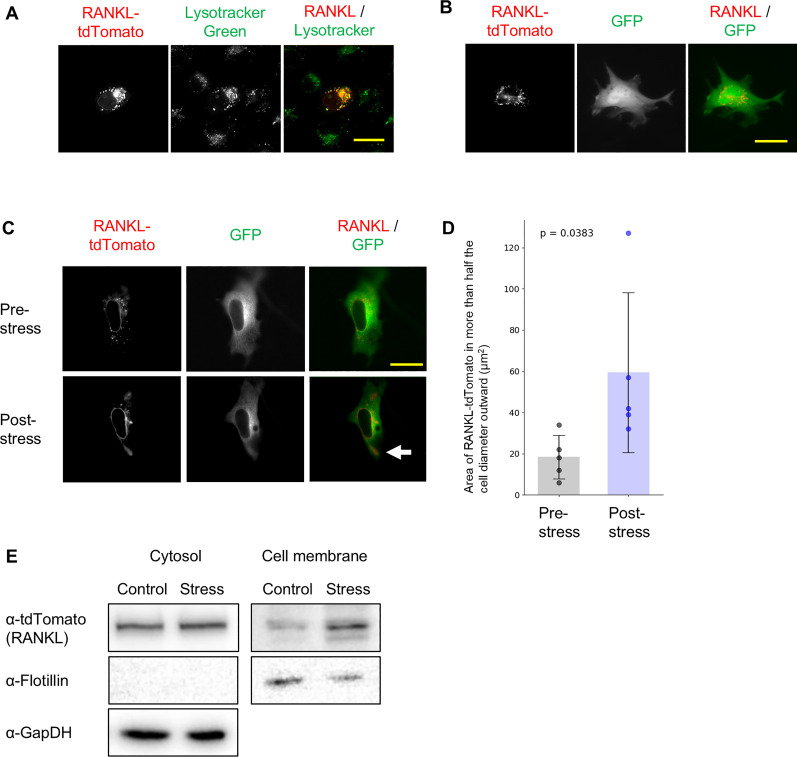
Fluid shear stress alters the localization of RANKL. (A) MC3T3-E1 cells were transfected with tdTomato-tagged RANKL (RANKL-tdTomato). After 48 h of culture, cells were treated with 50 nM LysoTracker Green DND-26 for 30 min and observed using a DeltaVision Elite microscope system. Scale bar: 30 μm. (B, C) MC3T3-E1 cells were co-transfected with RANKL-tdTomato and GFP. After 48 h of culture, cells were observed using the DeltaVision Elite system (B). Cells were subjected to fluid shear stress (12 dyn/cm²) for 10 min, and images were captured before and after stimulation. Representative images are shown (C). Scale bar: 30 μm. (D) The Area of RANKL-tomato in more than half the cell diameter outward was measured with Image J. The value for each sample is represented by a dot. The p-values are displayed on the graph. Data are presented as mean ± SEM (*n* = 5). (E) MC3T3-E1 cells were exposed to fluid shear stress (12 dyn/cm²) for 10 min, and proteins were extracted. Protein expression was analyzed by Western blotting.

First, we investigated whether fluid shear stress induces the localization of RANKL to the plasma membrane. When tdTomato-labeled RANKL was introduced into MC3T3-E1 cells, it was localized intracellularly and colocalized with lysosomal organelles labeled with LysoTracker (Fig. 1A and B). Upon application of fluid shear stress, the localization of RANKL shifted from the intracellular region to more than half the cell diameter outward (Fig. 1C and D). Consistent with the imaging results, Western blotting analysis of subcellular fractions revealed that fluid shear stress increased the amount of RANKL in the membrane fraction (Fig. 1E). These results strongly suggest that RANKL localization alters to the membrane fraction in response to fluid shear stress.

### RANKL colocalizes with c-Src and alters its localization upon fluid shear stress

**Fig. 2:**
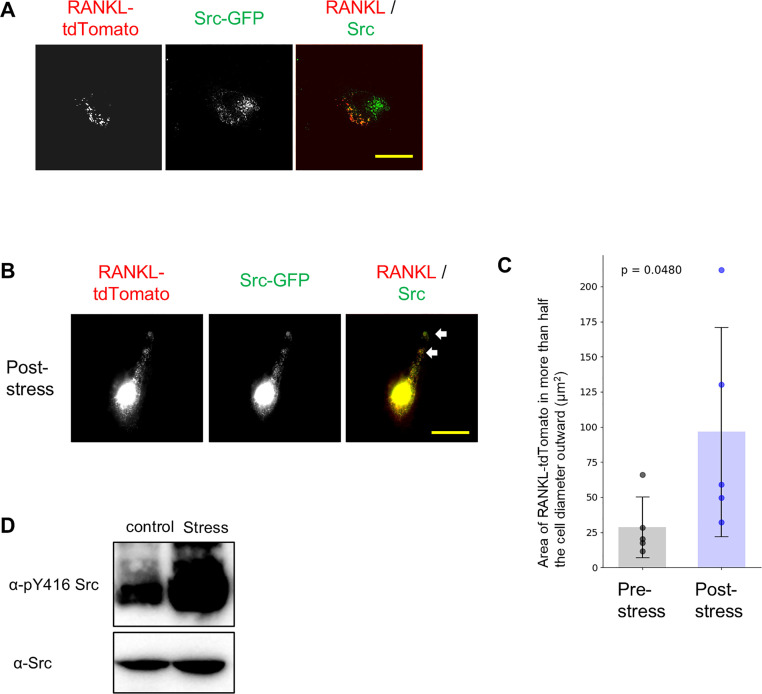
RANKL colocalizes with c-Src and alters the localization upon shear stress. (A) MC3T3-E1 cells were co-transfected with RANKL-tdTomato and Src-GFP. After 48 h of culture, cells were observed using the DeltaVision Elite system. Scale bar: 30 μm. (B, C) After 48 h of culture, cells were subjected to fluid shear stress (12 dyn/cm²) for 10 min and observed using the DeltaVision Elite system. Representative images are shown (B). The Area of RANKL-tomato in more than half the cell diameter outward was measured with Image J. The value for each sample is represented by a dot. The p-values are displayed on the graph. Data are presented as mean ± SEM (*n* = 5) (C). (E) MC3T3-E1 cells were exposed to fluid shear stress (12 dyn/cm²) for 10 min, and proteins were extracted. Protein expression was analyzed by Western blotting.

To investigate whether c-Src regulates RANKL localization, we examined the intracellular localization of c-Src. We found that c-Src colocalized with RANKL, and both proteins were localized in more than half the cell diameter outward upon exposure to fluid shear stress (Fig. 2A and B). Furthermore, fluid shear stress increased the phosphorylation of tyrosine 416 (Tyr416), an indicator of c-Src activation (Fig. 2C). These results suggest that mechanical stress-induced phosphorylation of c-Src may promote the localization change of RANKL to the plasma membrane.

### Activation of c-Src induces RANKL localization to the plasma membrane

**Fig. 3:**
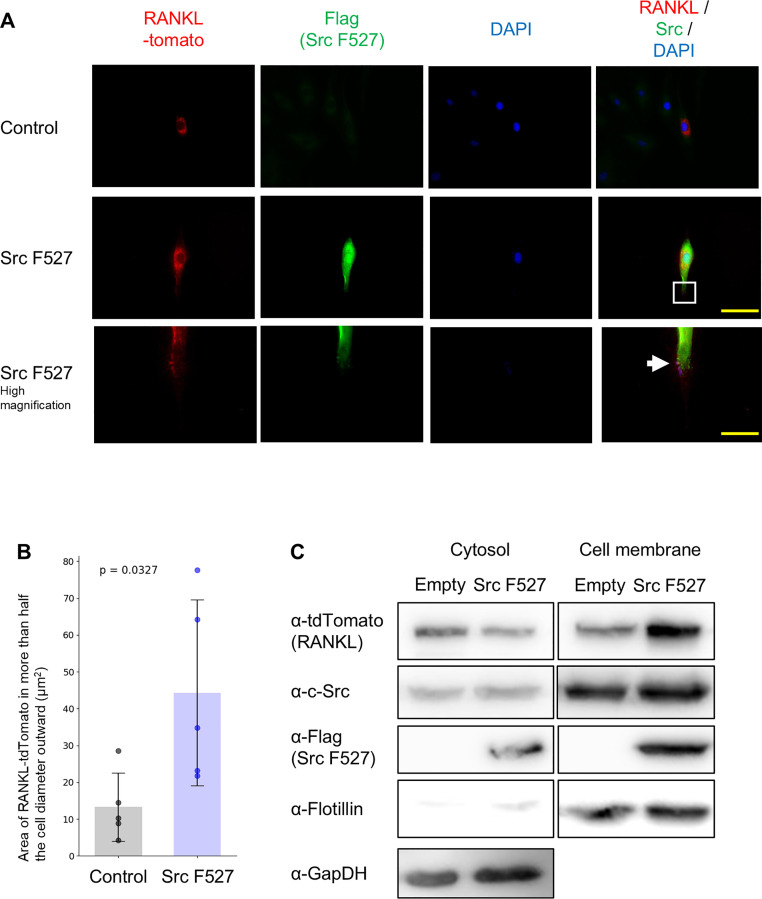
Activated c-Src promotes RANKL localization to the cell periphery. (A, B) MC3T3-E1 cells were co-transfected with RANKL-tdTomato and Src Y527F-Flag. After 48 h of culture, cells were fixed and analyzed by immunofluorescence staining. Scale bar: 30 μm (A). The Area of RANKL-tomato in more than half the cell diameter outward was measured with Image J. The value for each sample is represented by a dot. The p-values are displayed on the graph. Data are presented as mean ± SEM (*n* = 5) (B). (C) After 48 h of culture, proteins were extracted, and protein expression was analyzed by Western blotting.

To determine whether c-Src activation facilitates RANKL translocation to the plasma membrane, we performed experiments using a constitutively active c-Src mutant, Src Y527F. While RANKL was typically localized in the perinuclear region, overexpression of Src Y527F resulted in RANKL localization at more than half the cell diameter outward even in the absence of mechanical stress (Fig. 3A and 3B). Consistent with the imaging results, Western blotting confirmed that the amount of RANKL in the membrane fraction increased, even without mechanical stress (Fig. 3C). These findings indicate that c-Src, activated by mechanical stress, regulates the localization of RANKL to the membrane fraction.

## Discussion

In this study, we demonstrated that fluid shear stress activates c-Src and induces a shift in the intracellular localization of RANKL toward the plasma membrane. These findings suggest that mechanical stress may affect not only the expression levels of RANKL but also the process regulating its "surface presentation" to osteoclast precursors.

Furthermore, regarding parathyroid hormone (PTH), which promotes bone remodeling, it has been reported that its receptor, PTH1R, binds to c-Src in addition to signaling via canonical GPCR pathways, thereby activating downstream signals such as Erk [17]. This suggests the possibility that RANKL membrane translocation (surface presentation) is also regulated via c-Src activation under PTH stimulation. However, as the present study was limited to observations under mechanical stress conditions, the contribution of PTH to RANKL membrane translocation remains a subject for future investigation.

The effects of mechanical stress on bone tissue vary depending on the type, magnitude, and mode of loading [3,18]. For instance, in microgravity environments, bone resorption is accelerated, leading to bone loss [19]. Conversely, moderate mechanical loading, such as exercise, promotes bone formation [20]. In orthodontic treatment, strong local mechanical stress is applied to enhance bone resorption, thereby facilitating tooth movement [21].

In this study, using an experimental system overexpressing RANKL and c-Src, it was suggested that fluid shear stress alters the localization of RANKL, and that c-Src is involved in this alteration. Singh et al. demonstrated that membrane-localized RANKL possesses the ability to induce osteoclast differentiation upon stimulation with 1,25(OH)_2_D_3_ [22]. Based on this, it can be inferred that RANKL translocated to the membrane fraction by fluid shear stress also has osteoclast-inducing ability. Because fluid shear stress is a transient stimulus and the change in RANKL localization within this experimental system is also considered transient, demonstrating the complete process of osteoclast differentiation is difficult. Therefore, in future studies, employing an experimental system capable of measuring early signals of osteoclast differentiation via a luciferase assay—as utilized in the study by Singh et al. - may make it possible to demonstrate the relationship between the altered localization of RANKL and its ability to induce osteoclast differentiation. This point remains a subject for future investigation.

The mechanical stimulus used in this study was in vitro fluid shear stress, and it remains unclear how this corresponds to the loading magnitude or the dominant mechanical stimuli (fluid shear, compression, stretching, etc.) within bone tissue. Therefore, future studies are needed to examine the effects of mechanical stress on the intracellular trafficking and surface presentation of RANKL using loading models that better reflect the in vivo bone remodeling microenvironment.

## Funding

This work was supported by grants from 10.13039/501100001691Japan Society for the Promotion of Science (KAKENHI 25670870, 18K09509 and 25K22696 to T. M., 25K02826 and 25K22695 to S.K.) and 10.13039/501100011907Mishima Kaiun Memorial Foundation to T.M.

## Supporting material

This paper includes a Western blot figure that has not been cropped, which serves as supporting data.

## CRediT authorship contribution statement

**Takuma Matsubara:** Writing – review & editing, Writing – original draft, Visualization, Project administration, Methodology, Investigation, Funding acquisition, Formal analysis, Data curation, Conceptualization. **Anna Yoshimura:** Writing – review & editing, Validation. **Shoichiro Kokabu:** Writing – review & editing, Supervision, Funding acquisition.

## Declaration of competing interest

The authors declare that they have no known competing financial interests or personal relationships that could have appeared to influence the work reported in this paper.

## Data Availability

Data will be made available on request.

## Appendix Supplementary materials

Image, application 1

Image, application 2
