# Supplementary material for: Fluid shear stress activates c-Src and promotes RANKL localization in the plasma membrane in osteoblast-like MC3T3-E1 cells
Source: BBA Adv. 2026 Mar 20;9:100185. doi: 10.1016/j.bbadva.2026.100185 (PMC13054614; doi:10.1016/j.bbadva.2026.100185)
Supplement: Supplementary file 1 [file mmc1.docx]

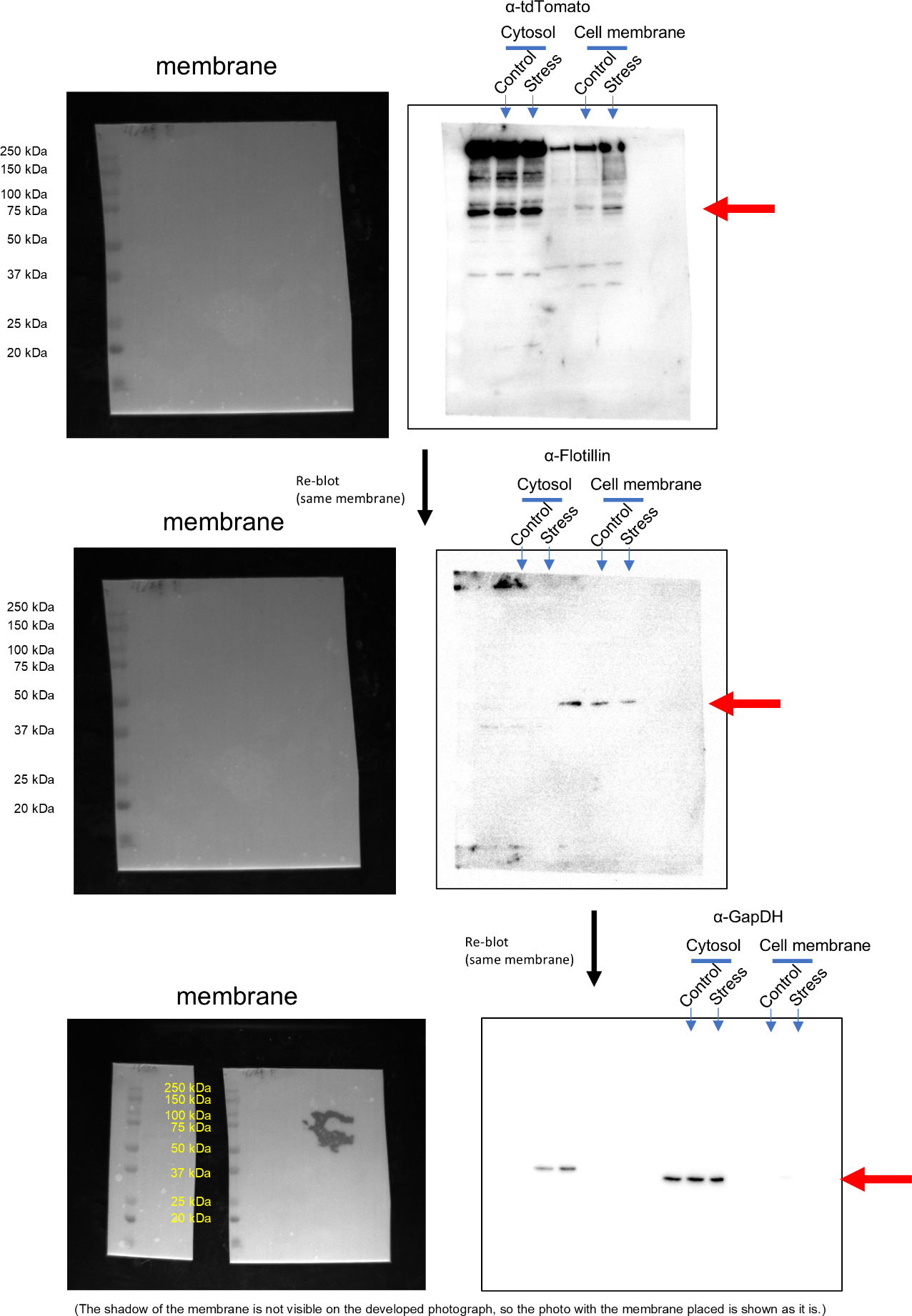


**Figure S1. Uncropped image of figure 1E**

The portion marked by the arrow has been cropped in the main figure.


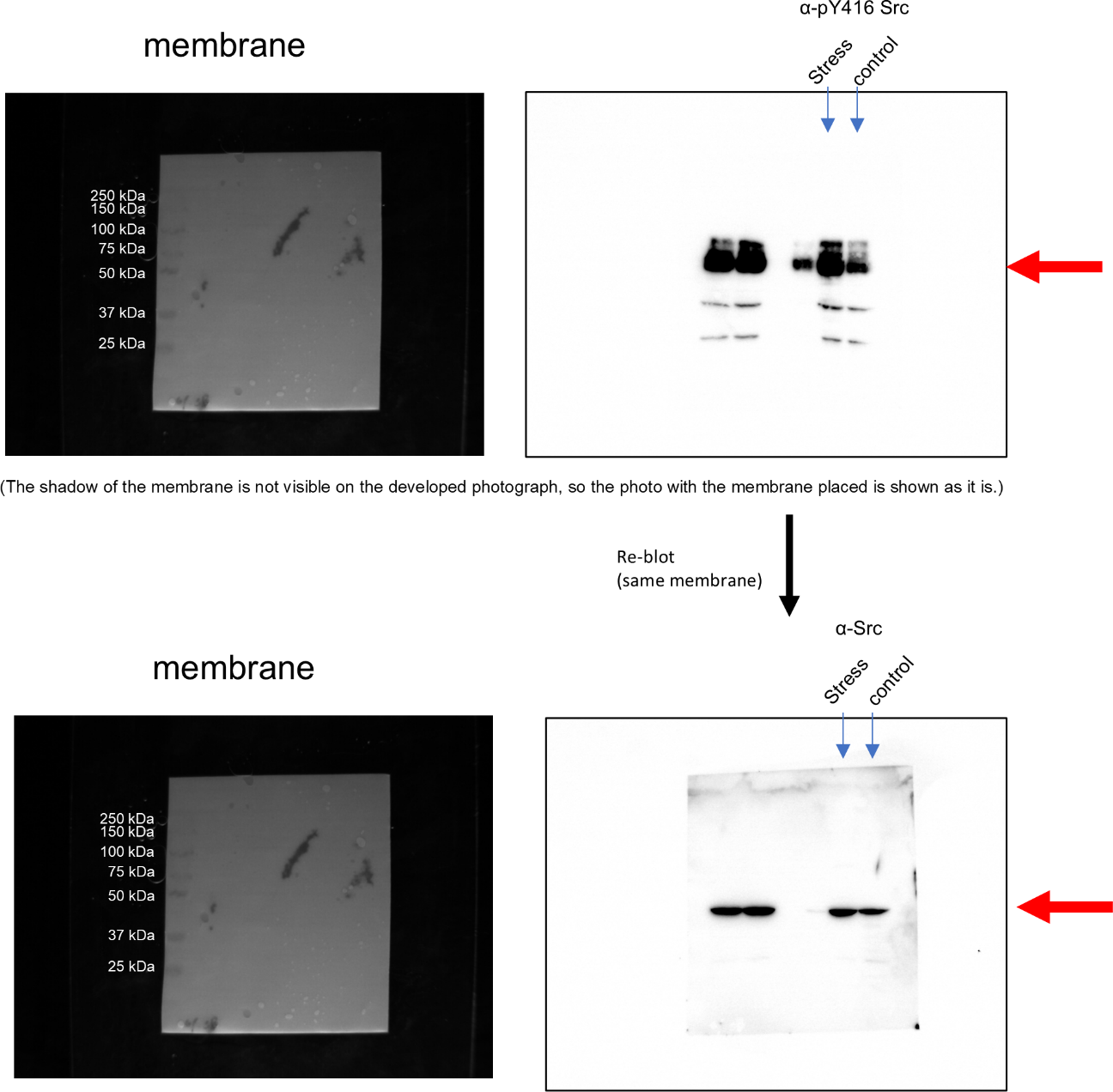


**Figure S2. Uncropped image of figure 2D**

The portion marked by the arrow has been cropped in the main figure.

Due to the order in which samples are run, they appear reversed left-to-right in the main figure.


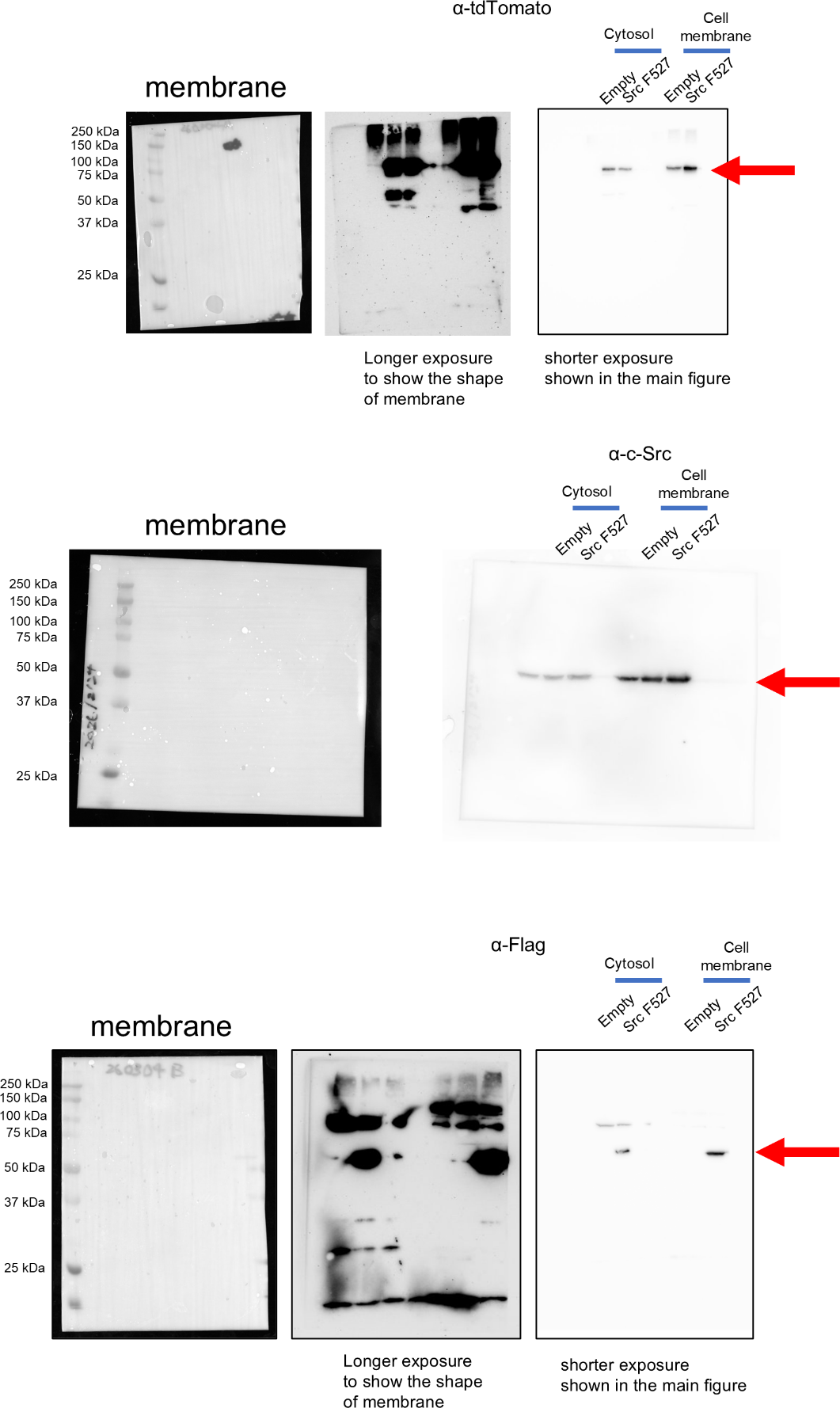


**Figure S3. Uncropped image of figure 3B**

The portion marked by the arrow has been cropped in the main figure.


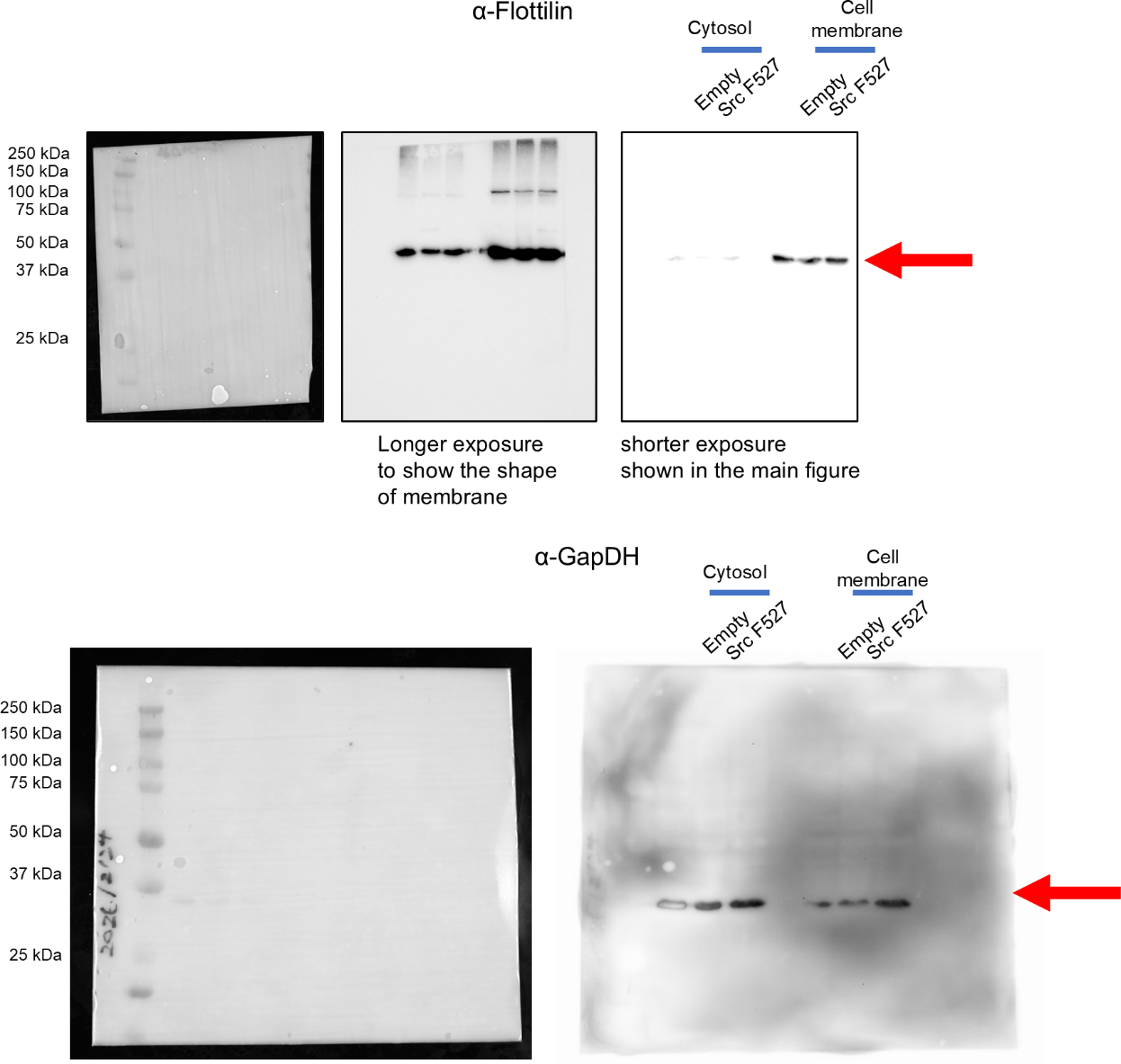


**Figure S4. Uncropped image of figure 3B**

The portion marked by the arrow has been cropped in the main figure.
