# Supplementary material for: Fluid shear stress activates c-Src and promotes RANKL localization in the plasma membrane in osteoblast-like MC3T3-E1 cells
Source: BBA Adv. 2026 Mar 20;9:100185. doi: 10.1016/j.bbadva.2026.100185 (PMC13054614; doi:10.1016/j.bbadva.2026.100185)
Supplement: Supplementary file 2 [file mmc2.pptx]

## Slide 1
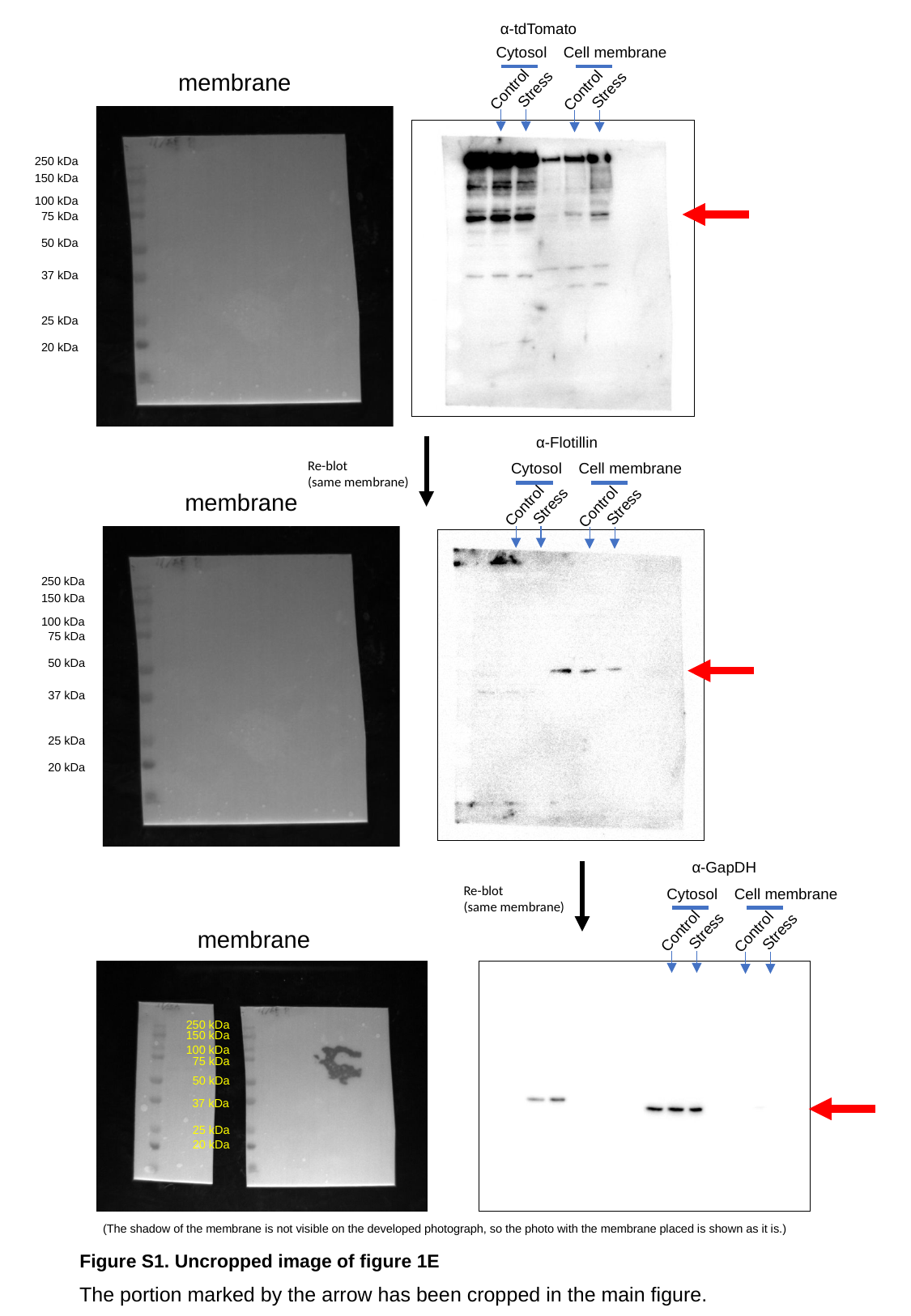

α-tdTomato
Cytosol
Cell membrane
membrane
Control
Stress
Control
Stress
250 kDa
150 kDa
100 kDa
75 kDa
50 kDa
37 kDa
25 kDa
20 kDa
α-Flotillin
Re-blot
(same membrane)
Cytosol
Cell membrane
membrane
Control
Stress
Control
Stress
250 kDa
150 kDa
100 kDa
75 kDa
50 kDa
37 kDa
25 kDa
20 kDa
α-GapDH
Re-blot
(same membrane)
Cytosol
Cell membrane
Control
Stress
Control
Stress
membrane
250 kDa
150 kDa
100 kDa
75 kDa
50 kDa
37 kDa
25 kDa
20 kDa
(The shadow of the membrane is not visible on the developed photograph, so the photo with the membrane placed is shown as it is.)
Figure S1. Uncropped image of figure 1E
The portion marked by the arrow has been cropped in the main figure.

## Slide 2
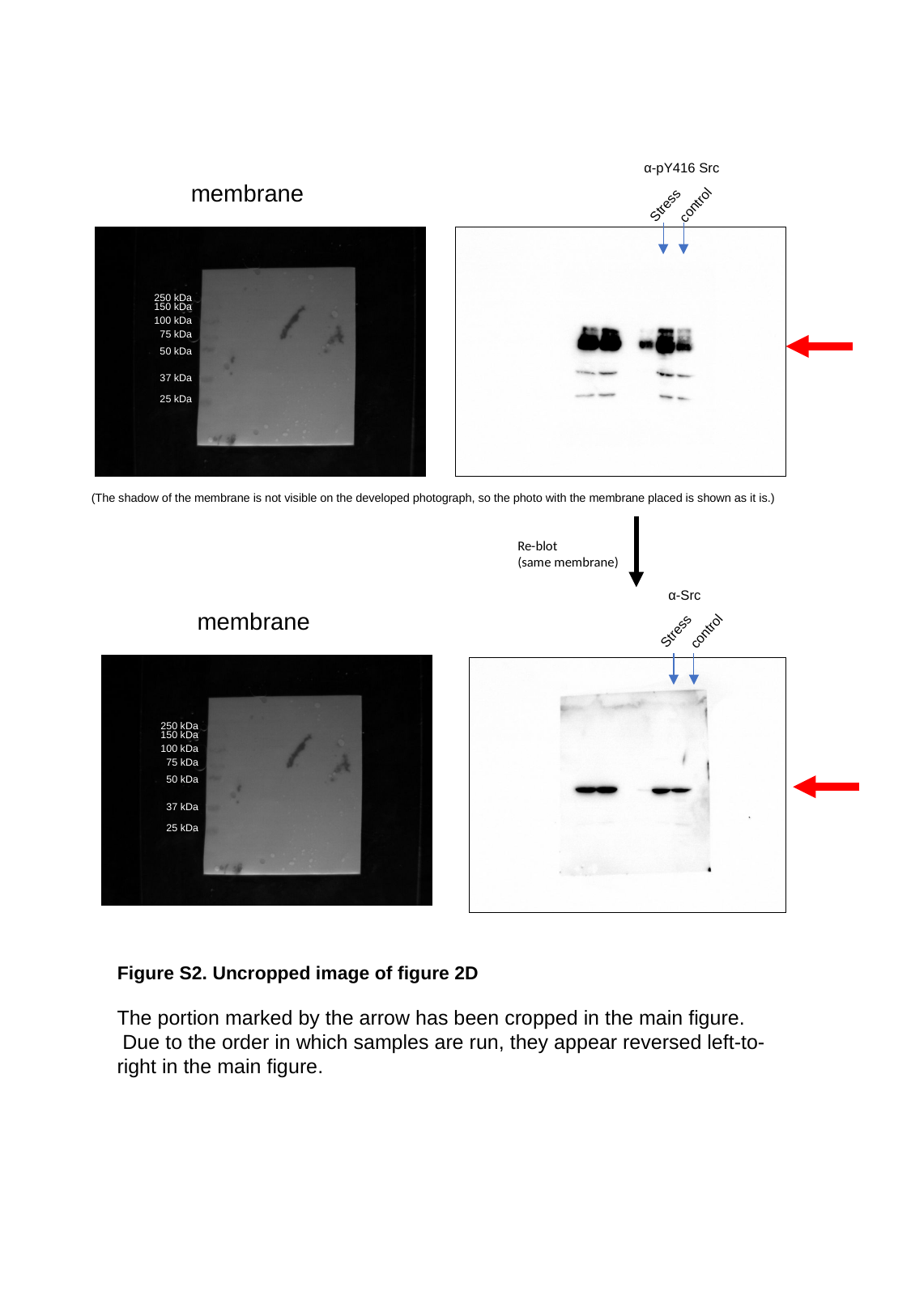

α-pY416 Src
membrane
Stress
control
250 kDa
150 kDa
100 kDa
75 kDa
50 kDa
37 kDa
25 kDa
(The shadow of the membrane is not visible on the developed photograph, so the photo with the membrane placed is shown as it is.)
Re-blot
(same membrane)
α-Src
membrane
Stress
control
250 kDa
150 kDa
100 kDa
75 kDa
50 kDa
37 kDa
25 kDa
Figure S2. Uncropped image of figure 2D
The portion marked by the arrow has been cropped in the main figure.
 Due to the order in which samples are run, they appear reversed left-to-right in the main figure.

## Slide 3
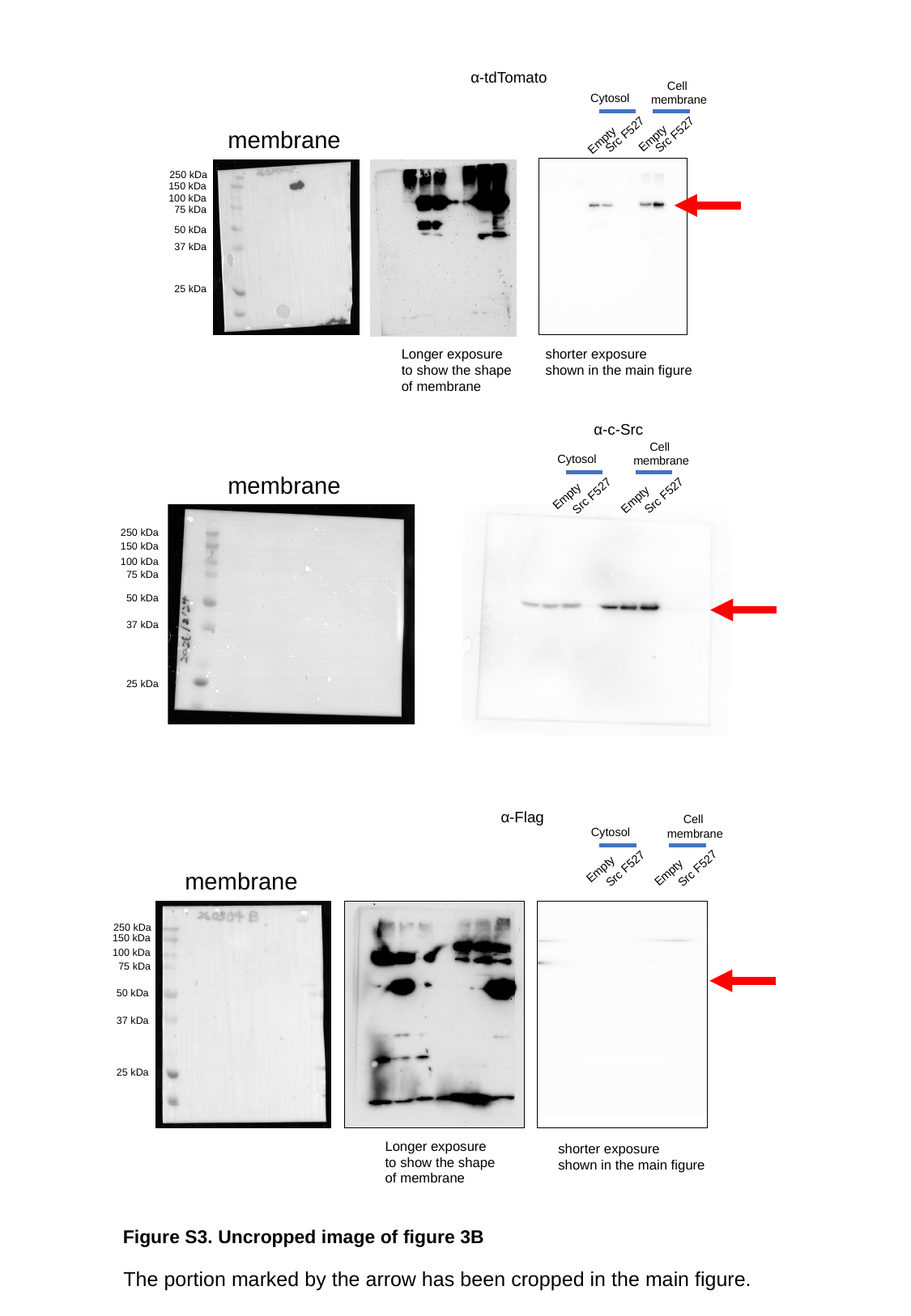

α-tdTomato
Cell
membrane
Cytosol
membrane
Src F527
Src F527
Empty
Empty
250 kDa
150 kDa
100 kDa
75 kDa
50 kDa
37 kDa
25 kDa
shorter exposure
shown in the main figure
Longer exposure
to show the shape
of membrane
α-c-Src
Cell
membrane
Cytosol
membrane
Src F527
Src F527
Empty
Empty
250 kDa
150 kDa
100 kDa
75 kDa
50 kDa
37 kDa
25 kDa
α-Flag
Cell
membrane
Cytosol
Src F527
Src F527
Empty
Empty
membrane
250 kDa
150 kDa
100 kDa
75 kDa
50 kDa
37 kDa
25 kDa
Longer exposure
to show the shape
of membrane
shorter exposure
shown in the main figure
Figure S3. Uncropped image of figure 3B
The portion marked by the arrow has been cropped in the main figure.

## Slide 4
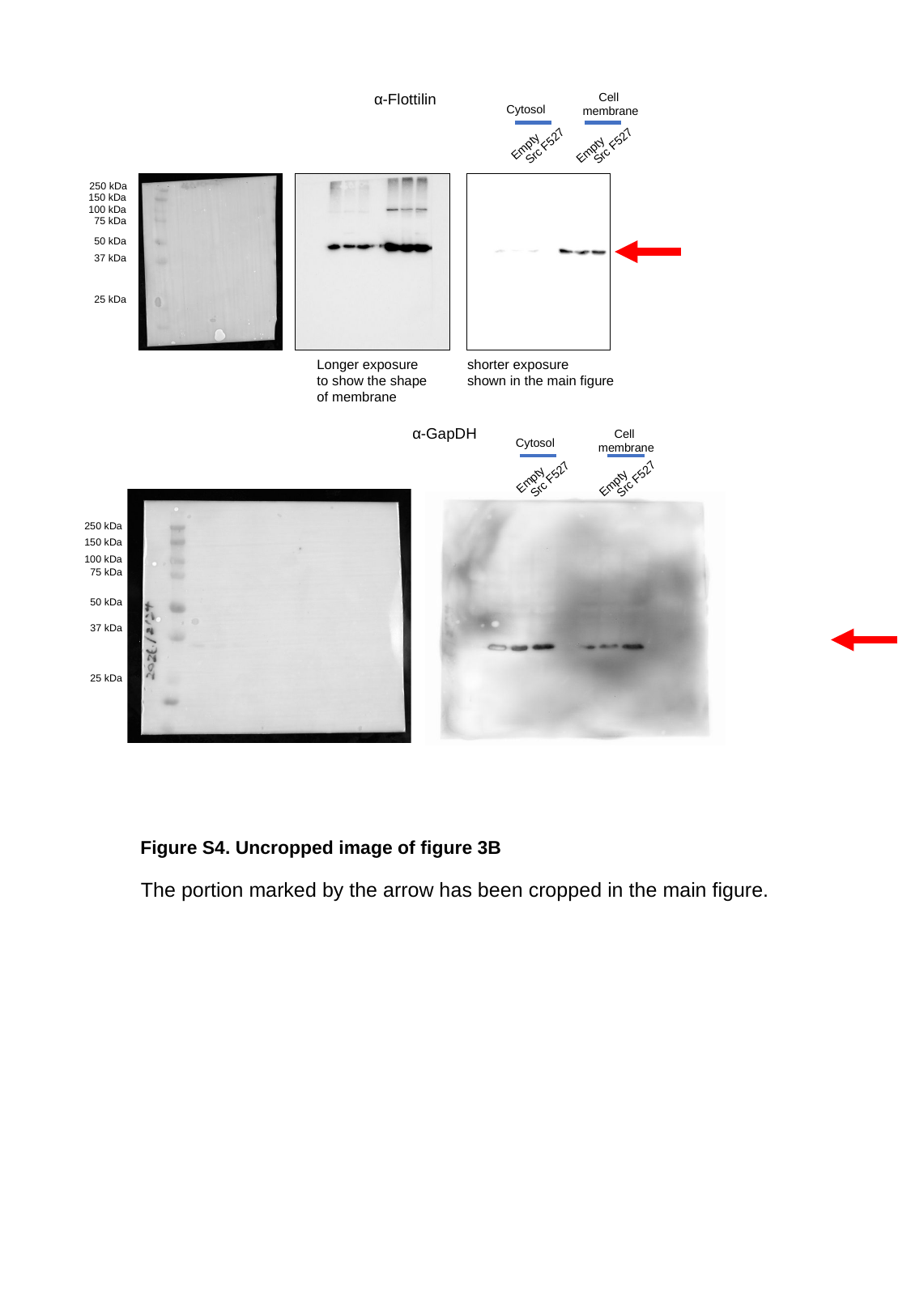

α-Flottilin
Cell
membrane
Cytosol
Src F527
Src F527
Empty
Empty
250 kDa
150 kDa
100 kDa
75 kDa
50 kDa
37 kDa
25 kDa
shorter exposure
shown in the main figure
Longer exposure
to show the shape
of membrane
α-GapDH
Cell
membrane
Cytosol
Src F527
Src F527
Empty
Empty
250 kDa
150 kDa
100 kDa
75 kDa
50 kDa
37 kDa
25 kDa
Figure S4. Uncropped image of figure 3B
The portion marked by the arrow has been cropped in the main figure.
